# Supplementary material for: Metabolic adaptations of micrometastases alter EV production to generate invasive microenvironments
Source: J Cell Biol. 2025 Jun 9;224(8):e202405061. doi: 10.1083/jcb.202405061 (PMC12147664; doi:10.1083/jcb.202405061)

File: SourceDataF7BD

MS: 202405061R

Metabolic adaptations of micrometastases alter EV production to generate invasive microenvironments

Figure 7 B&D

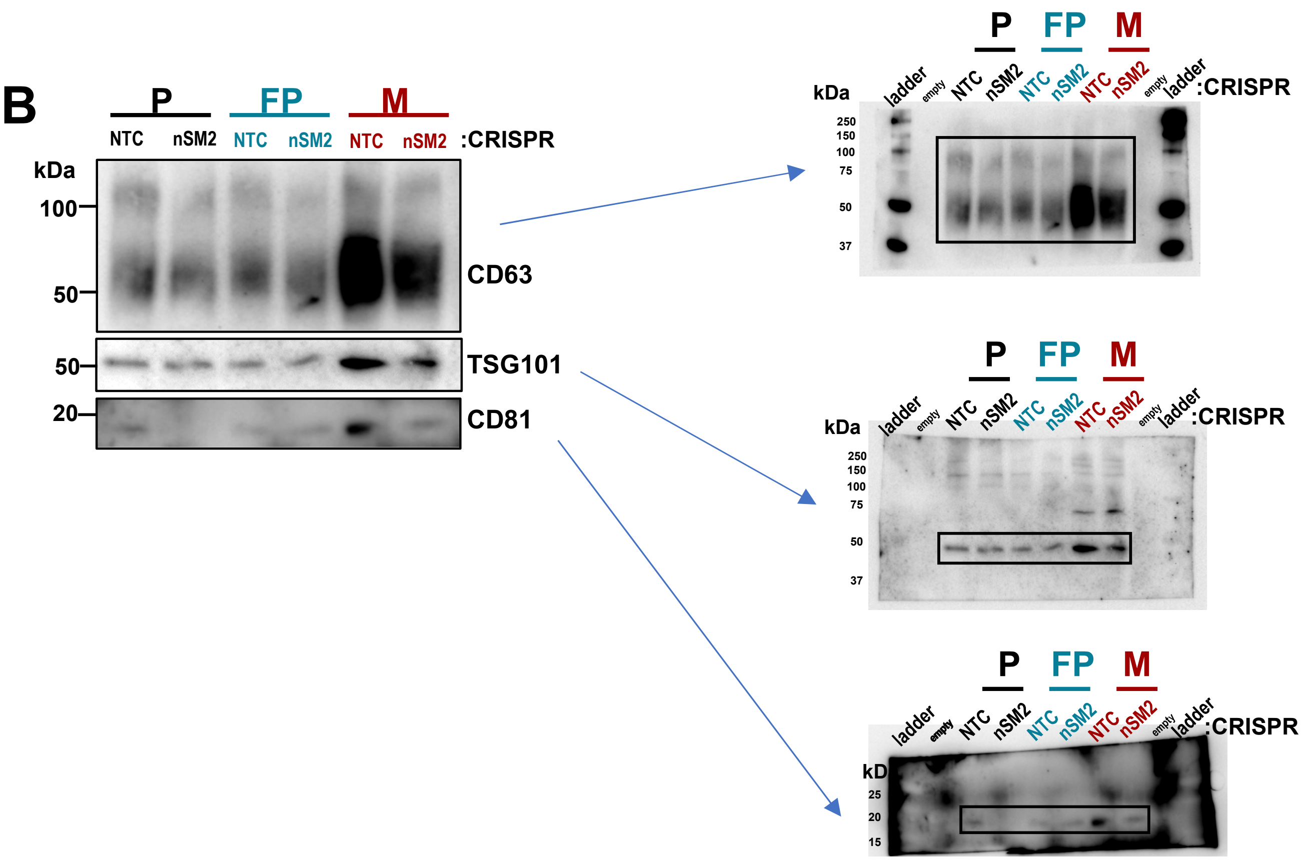

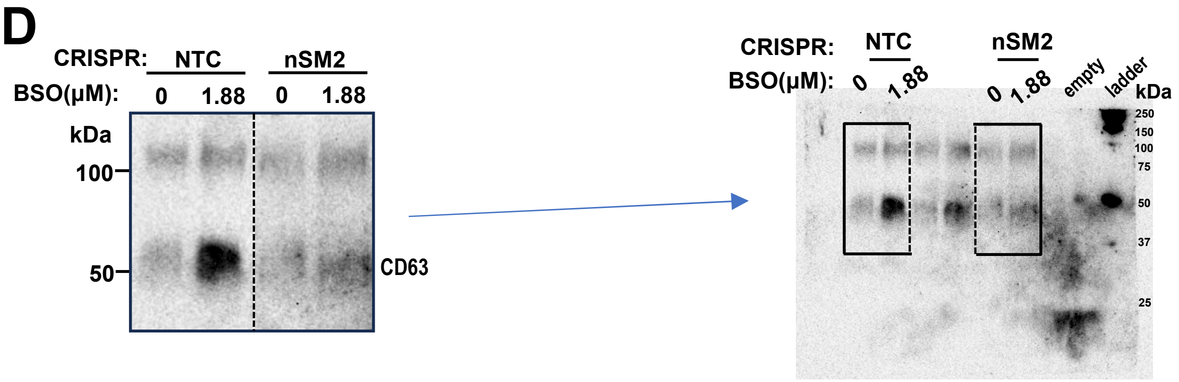

File: SourceDataF7F

MS: 202405061R

Metabolic adaptations of micrometastases alter EV production to generate invasive microenvironments

Figure 7F

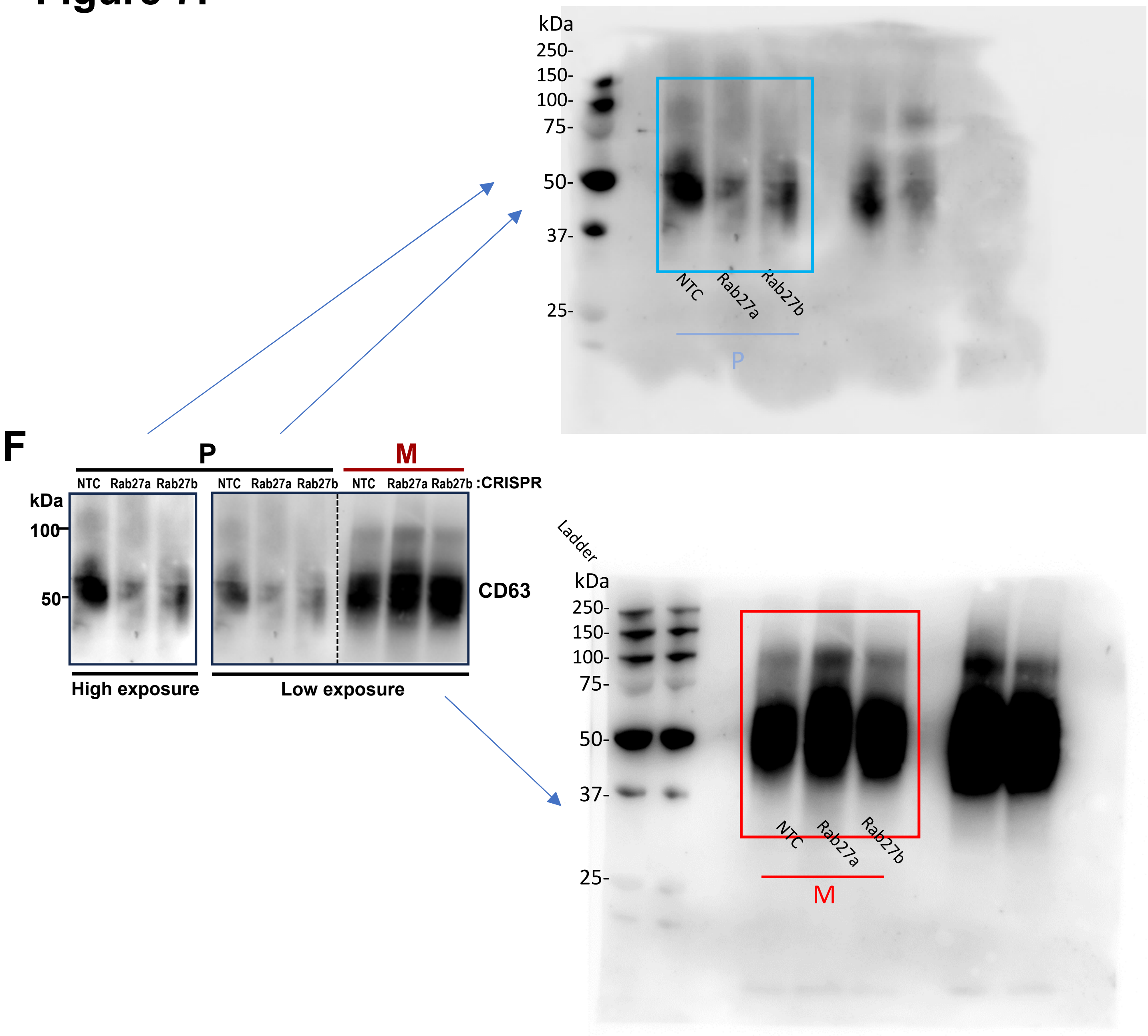

Supplement: SourceData F7 — is the source file for Fig. 7. [file jcb_202405061_sourcedataf7.pdf]
