# Supplementary material for: Metabolic adaptations of micrometastases alter EV production to generate invasive microenvironments
Source: J Cell Biol. 2025 Jun 9;224(8):e202405061. doi: 10.1083/jcb.202405061 (PMC12147664; doi:10.1083/jcb.202405061)

**File: SourceDataFS2D**

**MS: 202405061R**

Metabolic adaptations of micrometastases alter EV production to generate invasive microenvironments

# Figure S2D

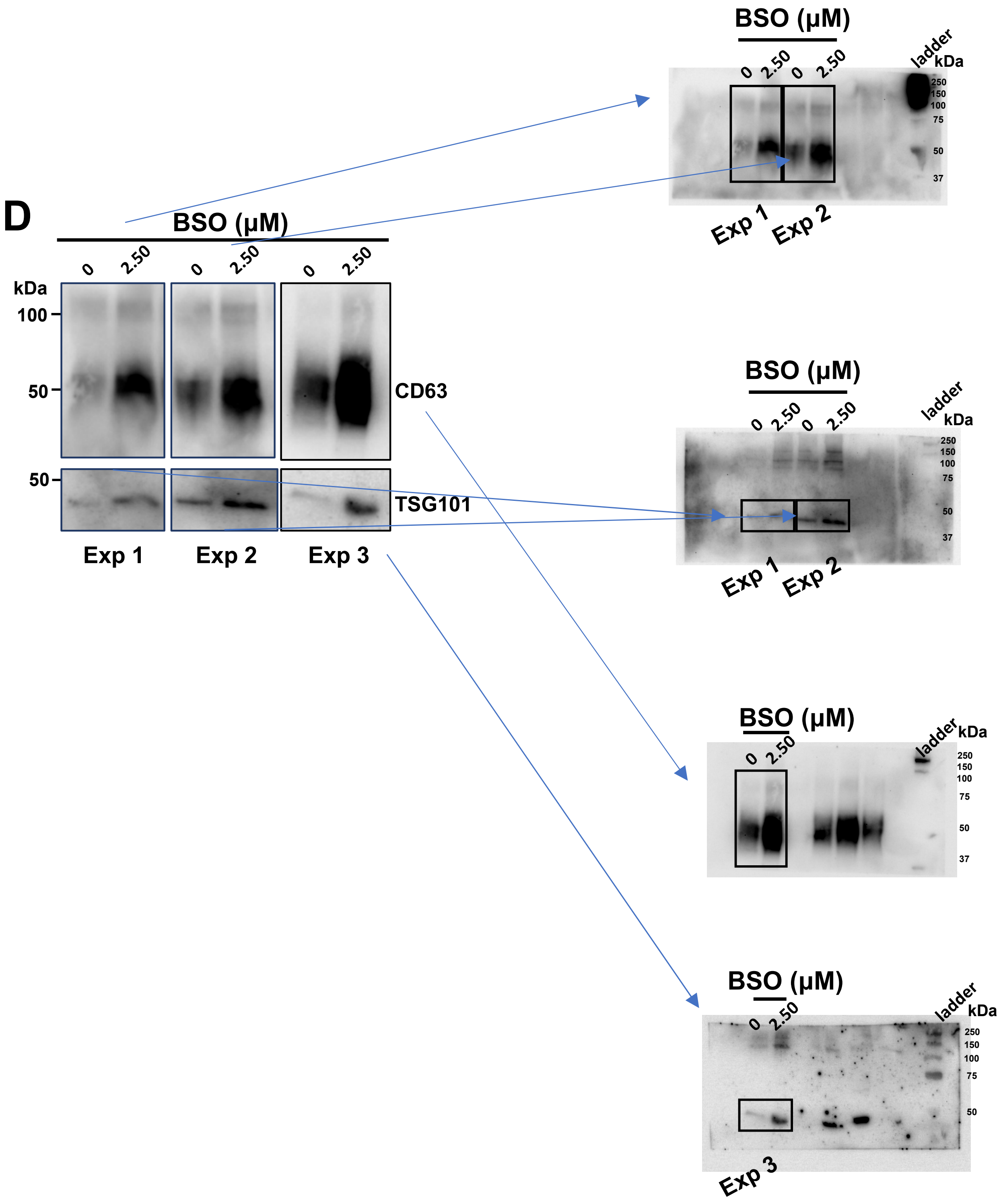

Supplement: SourceData FS2 — is the source file for Fig. S2. [file jcb_202405061_sourcedatafs2.pdf]
