# Supplementary material for: Metabolic adaptations of micrometastases alter EV production to generate invasive microenvironments
Source: J Cell Biol. 2025 Jun 9;224(8):e202405061. doi: 10.1083/jcb.202405061 (PMC12147664; doi:10.1083/jcb.202405061)

File: SourceDataFS3C

MS: 202405061R

Metabolic adaptations of micrometastases alter EV production to generate invasive microenvironments

Figure S3C

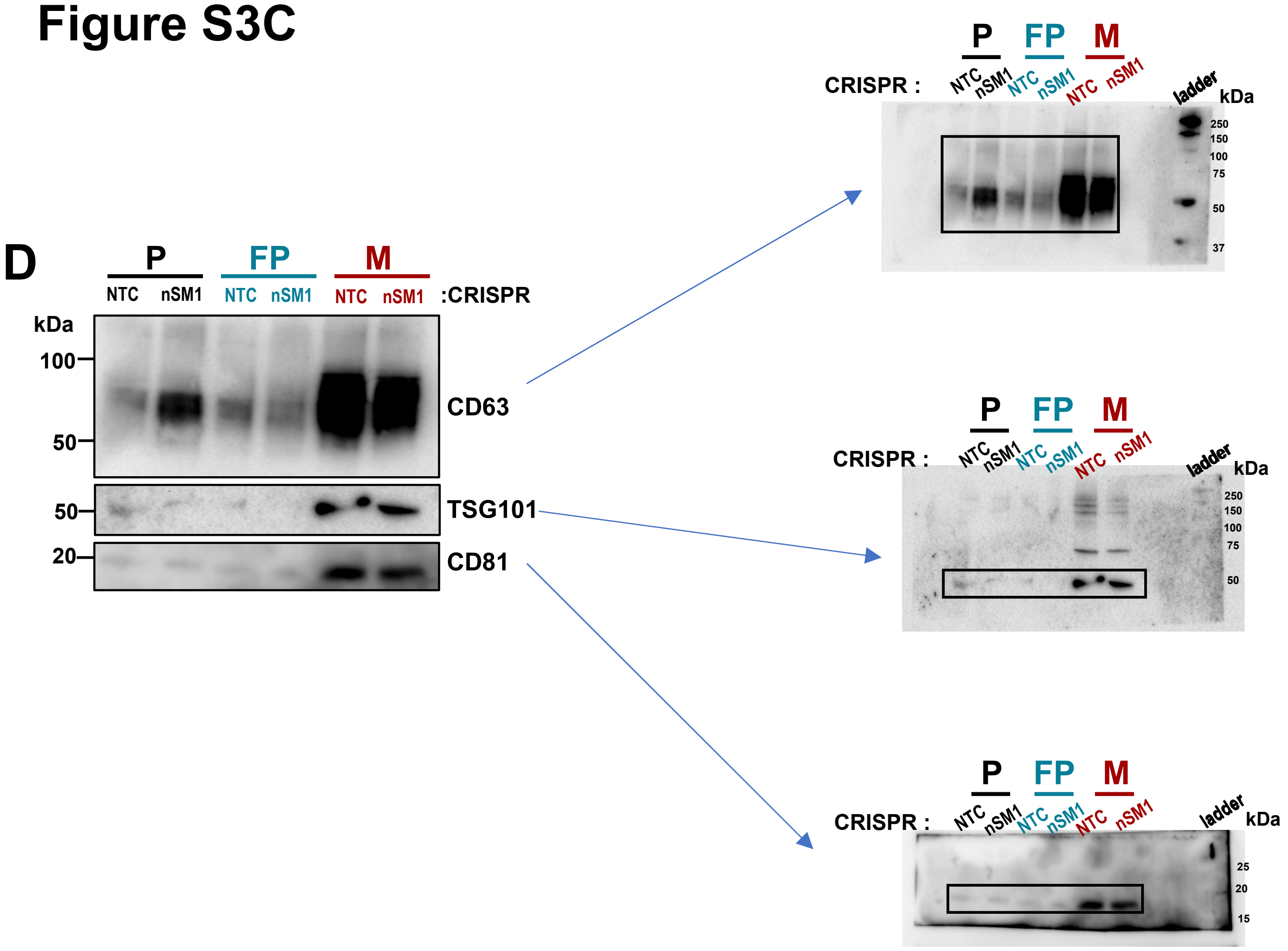

File: SourceDataFS3D

MS: 202405061R

Metabolic adaptations of micrometastases alter EV production to generate invasive microenvironments

Figure S3D

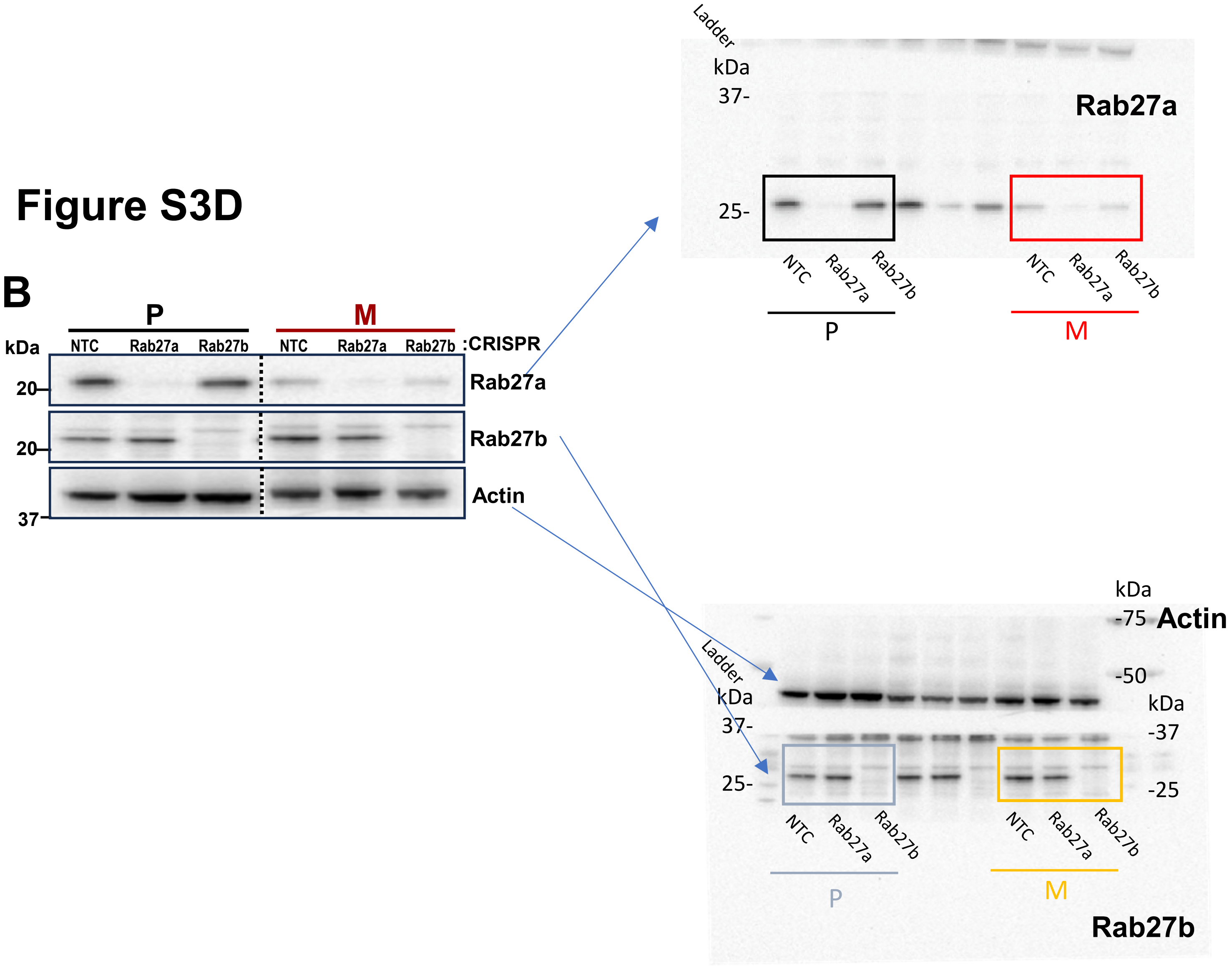

Supplement: SourceData FS3 — is the source file for Fig. S3. [file jcb_202405061_sourcedatafs3.pdf]
